# Deinoxanthin-Enriched Extracellular Vesicles from *Deinococcus radiodurans* Drive IL-10–Dependent Tolerogenic Programming of Dendritic Cells

**DOI:** 10.3390/antiox14091108

**Published:** 2025-09-12

**Authors:** Jeong Moo Han, Jaeyoon Lim, Woo Sik Kim, Bo-Gyeong Yoo, Jong-Hyun Jung, Sangyong Lim, Eui-Baek Byun

**Affiliations:** 1 Advanced Radiation Technology Institute, Korea Atomic Energy Research Institute, Jeongeup 56212, Republic of Korea; jmhahn@snu.ac.kr (J.M.H.); jylim94@kaeri.re.kr (J.L.);; 2 Department of Microbiology and Immunology, College of Medicine, Seoul National University, Seoul 03080, Republic of Korea; 3 Department of Food and Nutrition, Chungnam National University, Daejeon 34134, Republic of Korea; 4 Functional Biomaterial Research Center, Korea Research Institute of Bioscience and Biotechnology, Jeongeup 56212, Republic of Korea; kws6144@kribb.re.kr; 5 Department of Food Science and Technology, Kongju National University, Yesan 32439, Republic of Korea; 6 Department of Radiation Science, University of Science and Technology, Daejeon 34113, Republic of Korea

**Keywords:** Bacterial extracellular vesicles, *Deinococcus radiodurans*, Deinoxanthin, Dendritic cell maturation, Tolerogenic dendritic cells, Immune modulation, IL-10

## Abstract

Extracellular vesicles (EVs) derived from bacteria are emerging as potent bioactive carriers that affect host immunity. *Deinococcus radiodurans*, an extremophilic bacterium with strong antioxidant capacity, produces EVs enriched in deinoxanthin (DX), a carotenoid with a reactive oxygen species–scavenging activity. Here, we assessed the antioxidant activity of *D. radiodurans*-derived EVs (R1-EVs) in biochemical assays and their immunomodulatory effects on dendritic cells (DCs). R1-EVs exhibited significantly higher antioxidant activity than EVs from a DX-deficient mutant strain (ΔcrtI-EVs), consistent with DX enrichment. Bone marrow-derived DCs treated with R1-EVs in the presence of lipopolysaccharide displayed reduced expression of surface maturation markers and pro-inflammatory cytokines, while interleukin-10 (IL-10) production and antigen uptake were preserved, indicating a tolerogenic phenotype. This tolerogenic program led to decreased proliferation and cytokine production in allogeneic CD4^+^ and CD8^+^ T cells. Mechanistically, R1-EVs inhibited mitogen-activated protein kinase (MAPK) and nuclear factor kappa B (NF-κB) signaling pathways, key regulators of the DC activation. Importantly, IL-10 neutralization reversed these effects, restoring DC and T cell activation. Notably, ΔcrtI-EVs showed weaker antioxidant and immunoregulatory activities. Together, our findings identify R1-EVs as dual-functions, DX- and IL-10-dependent nanoplatform that integrates antioxidant and tolerogenic properties, with potential applications in inflammatory and autoimmune disease control.

## 1. Introduction

Extracellular vesicles (EVs) are nanoscale, lipid bilayer-enclosed structures secreted by organisms such as mammalian cells, plants, and bacteria. EVs have been shown to play a pivotal role in the process of intercellular act as key mediators of intercellular communication by transferring proteins, lipids, nucleic acids, and metabolites to recipient cells [1,2]. Considering their functional capabilities with regard to the transfer of bioactive components, EVs are being emphasized as a prospective therapeutic modality, with applications in domains such as immune modulation, drug delivery, and diagnostic procedures [3]. In recent years, bacterial extracellular vesicles (BEVs) have drawn considerable attention for their ability to modulate host responses, such as inflammation, oxidative stress, and immunity by delivering a broad range of bioactive cargo, including enzymes, membrane components, small RNAs, and metabolites that reflect the physiological characteristics of their parent cells [4,5]. For instance, Dhurve et al. found that *Acinetobacter baumannii* outer membrane vesicles (OMVs) are enriched with TonB-dependent iron transporters, which bind ferric-enterobactin and help shuttle iron into host cells [6]. In contrast, *Bacteroides fragilis* OMVs were shown to carry polysaccharide A, an immunomodulatory molecule that stimulates dendritic cells, leading to the activation of regulatory T cells and production of IL-10 [7]. Collectively, these studies suggest that the functional versatility of the BEVs is closely linked to the physiological and metabolic features of their parent strains, emphasizing the relevance of microbial origin in interpreting their effects on host systems [8,9].

*Deinococcus radiodurans* is a Gram-positive extremophilic bacterium known for its extraordinary resistance to ionizing radiation, desiccation, oxidative stress, and other environmental stresses [10,11]. This resilience is attributed to multiple coordinated defense strategies, such as highly efficient DNA repair mechanisms (e.g., extended synthesis-dependent strand annealing), protection of proteins against oxidative damage through manganese accumulation, robust antioxidant enzyme systems (e.g., catalase and superoxide dismutase), and the production of unique small molecules [12,13,14,15]. Among these protective molecules, the carotenoid deinoxanthin (DX) plays a notable role. The DX is a hydroxylated ketocarotenoid with potent reactive oxygen species (ROS), scavenging capacity and reflecting its intrinsic chemical antioxidant potential [16,17]. Beyond its intrinsic antioxidant capacity, the DX also provides cellular protection. In a UVB-induced skin damage model, it suppressed lipid peroxidation and boosted the superoxide dismutase (SOD) activity [18]. The DX, delivered via nanocapsules, further exhibited strong antioxidant and anti-inflammatory actions in vitro—reducing ROS and nitric oxide levels while improving stability and uptake by cells [19]. Together, these findings suggest that the DX not only supports the stress tolerance of *D. radiodurans* but may also help regulate immune and inflammatory responses in host systems.

We previously reported that EVs derived from *D. radiodurans* (R1-EVs) contain bioactive components, such as the DX and antioxidant enzymes, exhibiting potent antioxidant activity in oxidative stress models and radioprotective effects in vivo [20,21]. The immunological functions of R1-EVs have yet to be fully explored, especially in relation to innate and adaptive immunity. The specific contribution of the DX to their immunomodulatory activity also remains to be determined

To investigate whether the DX contributes to the immune-modulating effects of R1-Evs, we used bone marrow-derived dendritic cells (BMDCs), which are known to mediate both innate and adaptive immune responses [22]. We compared EVs from wild-type and DX-deficient *D. radiodurans* strains to assess their impact on the DC maturation, cytokine secretion, and downstream T-cell activation. This approach helped clarify how bacterial carotenoids like the DX influence host immunity.

## 2. Materials and Methods

### 2.1. Bacterial Strain and Culture Conditions

*Deinococcus radiodurans* R1 (ATCC 13939) was obtained from the American Type Culture Collection (ATCC, Manassas, VA, USA) and were cultured at 30 °C in tryptone glucose yeast extract (TGY) broth comprising 0.5% tryptone (Difco Laboratories, Detroit, MI, USA), 0.3% yeast extract (Difco Laboratories), and 0.1% glucose (Sigma–Aldrich, St. Louis, MO, USA) or on TGY plates with 1.5% Bacto-agar (Difco Laboratories). Antibiotics (8 μg/mL kanamycin; Sigma–Aldrich) were added to the medium, when it was necessary.

### 2.2. crtI Deletion Mutant Construction

A crtI (*dr_0861*) deletion mutant was constructed using fusion PCR, as previously described [23]. Briefly, a fusion PCR product for crtI deletion was generated in two steps using specific primer pairs and cloned into the pGEM^®^-T Easy vector (Promega, Madison, WI, USA), resulting in the plasmid pΔcrtI. A HincII-digested fragment containing a kanamycin resistance cassette derived from pKatAPH3 was subsequently inserted into the SmaI site of pΔcrtI, generating the recombinant plasmid pΔcrtI::kan. This recombinant plasmid was introduced into *D. radiodurans* as previously described [24]. Transformed *D. radiodurans* cells were selected on the TGY agar plates (0.5% tryptone, 0.3% yeast extract, 0.1% glucose) supplemented with kanamycin (8 μg/mL). The deletion of crtI was confirmed by the loss of red pigment. The primers used for deletion construction are listed in Table S1.

### 2.3. Isolation of BEVs

EVs were isolated from both wild-type *D. radiodurans* (R1-EVs) and the DX-deficient mutant strain of *D. radiodurans* (ΔcrtI-EVs) using the same protocol. Strains were cultured in the TGY broth at 30 °C under static conditions for 72 h. The culture supernatants were collected by centrifugation at 10,000× *g* for 30 min at 4 °C, followed by filtration through a 0.45 μm bottle-top vacuum filter system (Corning, Merck KGaA, Darmstadt, Germany). EVs were concentrated from 1 L to 25 mL using tangential flow filtration (TFF) equipped with a 500 kDa MWCO ultrafiltration membrane capsule (Pall Life Sciences, Port Washington, NY, USA), a cutoff commonly applied to retain nanosized vesicles while removing smaller contaminants [25]. The harvested retentate (25 mL) was carefully layered onto 5 mL of 60% iodixanol (Optiprep; StemCell Technologies, Vancouver, BC, Canada) in a 30 mL ultracentrifuge tube (Beckman Coulter, Brea, CA, USA), and cushioned ultracentrifugation was performed at 120,000× *g* for 90 min at 4 °C to isolate high-purity R1-EVs while minimizing protein contamination. The final EV pellets were resuspended in the PBS and stored at −80 °C. The protein content was quantified using a bicinchoninic acid protein assay kit (Thermo Scientific Pierce, Rockford, IL, USA) according to the manufacturer’s instructions.

### 2.4. Characterization of BEVs

The physicochemical properties of BEVs isolated from wild-type *D. radiodurans* (R1-EVs) and the DX-deficient mutant (ΔcrtI-EVs) were characterized by measuring particle size, zeta potential, and concentration using dynamic light scattering (DLS), transmission electron microscopy (TEM), and a nanoparticle tracking analysis (NTA).

#### 2.4.1. Particle Size and Zeta Potential Analysis

The BEVs were diluted in the PBS to an appropriate concentration and measured at 25 °C using a Zetasizer Nano ZS Zen3600 (Malvern Instruments, Malvern, UK). Measurements included a hydrodynamic diameter (mean particle size), the polydispersity index (PDI), and the zeta potential, which provided insight into the EV size distribution and surface charge.

#### 2.4.2. Transmission Electron Microscopy Analysis (TEM)

For ultrastructural visualization, the BEV samples were dispersed in ethanol and applied onto carbon-coated 150-mesh nickel grids, followed by air drying. The grids were imaged using a JEM-2100F (JEOL Ltd., Tokyo,, Japan) field emission TEM operated at 200 kV.

#### 2.4.3. Nanoparticle Tracking Analysis (NTA)

Particle concentration and size distribution were evaluated using a ZetaView NTA system (Particle Metrix, Meerbusch, Germany). The instrument was calibrated with 100 nm polystyrene standard particles (1:250,000 dilution; Applied Microspheres, Leusden, The Netherlands). Samples were equilibrated at room temperature for 20 min and diluted in the PBS or distilled water to achieve 140–200 particles per frame in a final volume of 1 mL. Measurements were conducted at 25 °C and pH 7.0 under default EV settings (autofocus, camera sensitivity 75, shutter speed 100). For each sample, three cycles were recorded across 11 cell positions with 30 frames per position. The ZetaView Software 8.04.02 SP2 was used for data analysis with preset parameters (maximum area: 1000, minimum area: 5, minimum brightness: 25), ensuring a minimum of 1000 completed tracks per sample.

### 2.5. Antibodies and Reagents

The primary BMDC cultures were differentiated using the recombinant mouse granulocyte-macrophage colony-stimulating factor (rmGM-CSF) and interleukin-4 (rmIL-4) (JW CreaGene, Seongnam,, Korea). Ultrapure lipopolysaccharide (LPS) from *E. coli* O111:B4 (Invivogen, San Diego, CA, USA) was used as a stimulant for in vitro maturation of BMDCs. Annexin V and propidium iodide (PI) staining kits were purchased from BD Biosciences (Milpitas, CA, USA) for cytotoxicity analysis. Fluorescence-conjugated antibodies (Abs) for flow cytometry analysis included: Live/Dead Cell Staining Kit (L/D; BV510; Invitrogen, Carlsbad, CA, USA); Cell Proliferation Dye (CPD) eFluor 450; anti-MHC-I (APC), MHC-II (PE-Cy7), CD3 (APC-Cy7), CD4 (Alexa488), CD8α (PerCP-Cy5.5), IFN-γ (PE), TNF-α (APC), IL-2 (PE-Cy7) (eBioscience, San Diego, CA, USA); and anti-CD11c (PE-Cy7), CD80 (FITC), CD86 (PE), TNF-α (APC), IL-12p70 (PE), IL-10 (FITC) (BD Biosciences). Isotype controls used for surface marker analysis included rat IgG2 kappa (FITC), rat IgG2a kappa (PE, APC), and rat IgG2b kappa (PE-Cy7) (BD Biosciences). For cytokine quantification, the ELISA kits, specific for mouse TNF-α, IL-12p70, IL-10, IFN-γ, IL-5, and IL-2, were obtained from BD Biosciences. Antigen uptake was assessed using the FITC-conjugated dextran (40,000 Da; Sigma-Aldrich). For immunoblotting and signaling analysis, antibodies against phosphorylated and total ERK, JNK, p38, IκB-α, NF-κB, lamin B, and β-actin were purchased from Cell Signaling Technology (Boston,, MA, USA). Naïve CD4^+^ and CD8^+^ T cells were isolated using the MACS MicroBead system with CD4^+^ and CD8^+^ T cell isolation kits (Miltenyi Biotec, Gaithersburg, MD, USA).

### 2.6. Ethics Statement and Mice

Seven-week-old female C57BL/6 and BALB/c mice (Orient Bio Inc., Seoul, Korea) were used for different experimental purposes, including the C57BL/6 mice were used for the differentiation of the BMDCs, and the BALB/c mice were used for the isolation of splenocytes. All animal procedures were conducted in accordance with the institutional guidelines of the Korea Atomic Energy Research Institute (KAERI, Jeongeup, Korea) and approved by the Institutional Animal Care and Use Committee (IACUC) of KAERI (Approval No. KAERI-IACUC-2024–010).

### 2.7. Treatment of BMDCs with R1-EVs and ΔcrtI-EVs

The bone marrow cells were flushed from the femurs and tibias of the C57BL/6 mice and treated with a red blood cell lysis buffer (Sigma–Aldrich). The remaining cells were cultured in the RPMI-1640 medium (Biowest, Nuaillé, France), supplemented with a 10% heat-inactivated fetal bovine serum (FBS), a 1% penicillin/streptomycin (P/S, Gibco, Carlsbad, CA, USA), a 20 ng/mL GM-CSF, and a 0.5 ng/mL IL-4 for eight days to generate the BMDCs. After differentiation, the cells were harvested and confirmed to contain > 90% CD11c^+^ cells by flow cytometry (FACSVerse, BD Bioscience) using an anti-CD11c antibody. After differentiation, the BMDCs (0.5 × 10^6^ cells/well in a 48-well plate) were incubated for 18 h under the following four conditions: untreated, the LPS alone (100 ng/mL), the LPS plus R1-EVs (5, 10, or 20 μg/mL), or the LPS plus ΔcrtI-EVs (same doses). The cells were cultured at 37 °C in a 5% CO_2_ incubator and analyzed for maturation markers or cytokine production as indicated.

### 2.8. Annexin V and Propidium Iodide (PI) Staining

The BMDCs treated with R1-EVs or ΔcrtI-EVs for 18 h were harvested and resuspended in Annexin V binding buffer (BD Bioscience). The cells were stained with Annexin V (1:50 dilution) for 15 min at room temperature (RT), washed, and subsequently stained with PI (1:25 dilution) for 10 min at RT. The cell death was quantified by flow cytometry (FACSverse, BD Bioscience) based on Annexin V^+^ (apoptotic), PI^+^ (necrotic), and Annexin V^+^PI^+^ (late apoptotic) populations. The data were analyzed using the FlowJo software (v10, BD Bioscience).

### 2.9. Analysis of Surface Molecules on BMDCs

To evaluate the suppressive effect of R1-EVs and ΔcrtI-EVs on LPS-induced DC maturation, the BMDCs were co-treated with the LPS (100 ng/mL) alone or in combination with R1-EVs or ΔcrtI-EVs (5, 10, or 20 μg/mL) for 18 h. After treatment, the cells were harvested and stained with L/D and fluorescence-conjugated antibodies against CD80, CD86, MHC-I, and MHC-II for 20 min at RT. The cells were then washed with a cold FACS buffer (PBS containing 2% FBS and 0.01% sodium azide) and fixed using an IC fixation buffer (eBioscience). Surface molecule expression was analyzed using a FACSVerse flow cytometer (BD Biosciences), and the data were processed with the FlowJo software (version 10).

### 2.10. Measurement of Extracellular Cytokine Levels

To assess cytokine secretion, culture supernatants were collected from the BMDCs treated with LPS (100 ng/mL) alone or in combination with R1-EVs or ΔcrtI-EVs (5, 10, or 20 μg/mL) for 18 h, as described in Section 2.7. The levels of TNF-α, IL-12p70, and IL-10 were quantified using mouse-specific ELISA kits (BD Biosciences), following the manufacturer’s protocols. Absorbance was measured at 450 nm using a microplate ELISA reader (Zenyth 3100, Anthos Labtec Instruments GmbH, Salzburg, Austria).

### 2.11. Detection of the Levels of Intracellular Cytokines in BMDCs

The BMDCs were stimulated with the LPS (100 ng/mL) alone or in combination with R1-EVs or ΔcrtI-EVs (5, 10, or 20 μg/mL) in the presence of GolgiPlug (BD Biosciences) for 8 h. The untreated and LPS-only groups served as controls. The cells were harvested, washed with a cold FACS buffer, and stained with Live/Dead and anti-CD11c antibodies for 20 min at RT. After surface staining, the cells were fixed and permeabilized using BD Cytofix/Cytoperm buffer for 20 min at 4 °C, followed by washing with BD Perm/Wash buffer. Intracellular staining was then performed using fluorescence-conjugated antibodies against TNF-α, IL-12p70, and IL-10. After two additional washes with the BD Perm/Wash buffer, cytokine levels in CD11c^+^ BMDCs were analyzed by flow cytometry using a FACSVerse instrument and the FlowJo software.

### 2.12. Analysis of the Antigen-Uptake Ability of BMDCs

The BMDCs were treated with the LPS (100 ng/mL) alone or in combination with R1-EVs or ΔcrtI-EVs (5, 10, or 20 μg/mL) for 18 h, followed by incubation with an FITC-conjugated dextran (1 mg/mL; 40,000 Da, Sigma–Aldrich) for 40 min in parallel at 37 °C and 4 °C. After incubation, the cells were washed three times with a cold FACS buffer, stained with anti-CD11c antibody for 20 min at RT, and washed again. Dextran uptake by CD11c^+^ cells was assessed using a FACSVerse cytometer and the FlowJo software. The 4 °C condition served as a control for passive adsorption, allowing discrimination of active antigen uptake at 37 °C.

### 2.13. Western Blotting Analysis

The BMDCs were treated with one of the following: LPS alone (100 ng/mL), LPS plus R1-EVs (20 μg/mL), or LPS plus ΔcrtI-EVs (20 μg/mL). The cells were harvested at the indicated time points (0, 15, 30, and 60 min) after stimulation. The cytosolic and nuclear protein fractions were isolated using an RIPA buffer (Pierce, Rockford, IL, USA) and the CelLytic NuCLEAR Extraction Kit (Sigma–Aldrich), respectively, according to the manufacturer’s instructions. Western blotting was performed as previously described [26].

### 2.14. Allogeneic Mixed Lymphocyte Reaction

Naive CD4^+^ and CD8^+^ T cells were isolated from the spleens of BALB/c mice using MACS CD4^+^ and CD8^+^ isolation kits. The cells were labeled with 1 μM Cell Proliferation Dye (CPD) eFluor 450 for 15 min at 37 °C in the dark, followed by three washes with cold PBS containing 10% FBS. The labeled T cells (5 × 10^5^ cells/well in 96-well plates) were co-cultured for three days with BMDCs (1 × 10^5^ cells/well) that had been subjected to different pre-treatments: untreated, LPS (100 ng/mL) alone, R1-EVs or ΔcrtI-EVs (20 μg/mL) alone, or the LPS in combination with either type of EV. The co-culture was carried out in RPMI-1640 medium supplemented with 10% FBS and 1% penicillin/streptomycin at 37 °C under 5% CO_2_. After the incubation period, the cell culture supernatants were collected to assess cytokine secretion (IFN-γ, IL-5, and IL-2) via ELISA. The T cells were harvested, stained with the anti-CD4 and anti-CD8 antibodies, and analyzed using a FACSVerse cytometer and the FlowJo software to determine proliferation rates of CD4^+^ and CD8^+^ T cells.

### 2.15. Inhibition of IL-10 by Neutralizing Antibody

To block IL-10 signaling, the BMDCs were pretreated with a 5 ng/mL of anti-mouse IL-10 neutralizing antibody (clone JES5-2A5; Bio X Cell, Lebanon, NH, USA) or a rat IgG1 isotype control for 2 h prior to stimulation with EVs or LPS.

### 2.16. Statistical Analysis

The data were analyzed using unpaired *t*-tests for two-group comparisons or one-way ANOVA followed by Tukey’s post hoc test for multiple group comparisons using GraphPad Prism 10 (GraphPad Software, San Diego, CA, USA). The results are expressed as the mean ± standard deviation (SD). Statistical significance was considered at *p < 0.05*. Significance indicators are defined in the respective figure legends.

## 3. Results

### 3.1. Isolation and Characterization of EVs Derived from Deinococcus radiodurans (R1-EVs) and ΔcrtI Mutant D. radiodurans (ΔcrtI-EVs)

**Figure 1:**
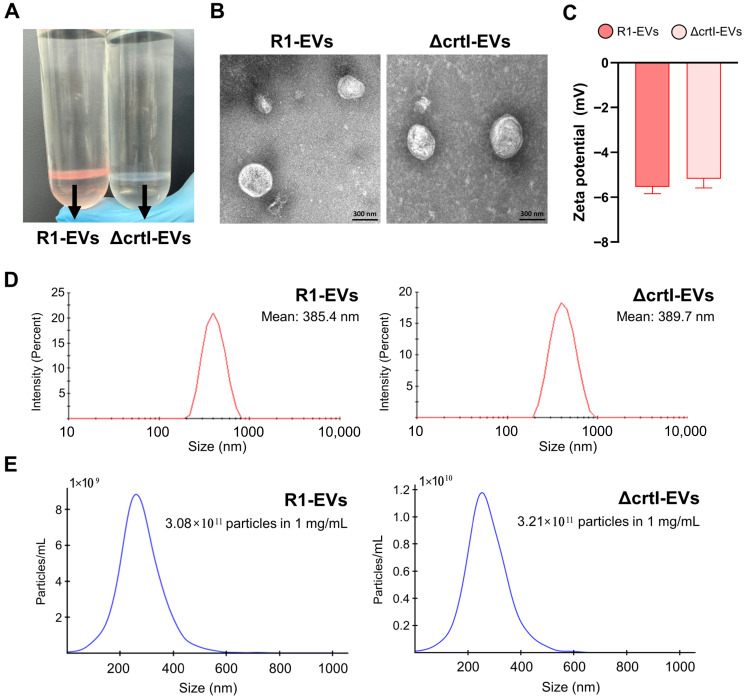
Morphological and physicochemical characterization of extracellular vesicles (EVs) derived from wild-type (R1-EVs) and ΔcrtI-mutant *D. radiodurans (*ΔcrtI-EVs). (**A**) OptiPrep gradient after EV purification showing a pink band for R1-EVs and a colorless band for ΔcrtI-EVs, consistent with the DX-associated pigmentation. (**B**) Transmission electron microscopy (TEM) image illustrating the spherical morphology of EVs. (**C**) Zeta potential measurement of EVs. (**D**) Dynamic light scattering (DLS) analysis showing the size distribution profile of EVs. (**E**) Nanoparticle tracking analysis (NTA) showing particle size and concentration.

EVs were successfully isolated from the culture supernatants of both wild-type *D. radiodurans* (R1) and the DX-deficient mutant (ΔcrtI-EVs) using a combination of differential centrifugation, TFF, and density gradient ultracentrifugation. The isolation procedure was conducted in accordance with the Minimal Information for Studies of EVs (MISEV) guidelines [27], and the resulting EVs were subjected to comprehensive physicochemical characterization. EVs were isolated from wild-type and ΔcrtI (DX-deficient) strains. During density-gradient purification, the R1-EV fraction displayed a characteristic pink hue, consistent with the DX pigmentation in the source strain, whereas the ΔcrtI-EV fraction was essentially colorless (Figure 1A). The TEM images showed that both EV types exhibited typical spherical (Figure 1B). Zeta potential measurements showed slightly negative surface charges for both EV populations, with no statistically significant difference between them (Figure 1C). DLS analysis revealed that the hydrodynamic diameters of R1-EVs and ΔcrtI-EVs were comparable, averaging 385.4 nm and 389.7 nm, respectively (Figure 1D). The NTA confirmed similar particle concentrations for both EV types, with 3.08 × 10^11^ particles/mL for R1-EVs and 3.21 × 10^11^ particles/mL for ΔcrtI-EVs in 1 mg/mL protein preparations (Figure 1E). The comparable morphology and physicochemical properties of R1-EVs and ΔcrtI-EVs indicate that deletion of dr0861 does not significantly affect the basic biophysical characteristics of the EVs, supporting their use in subsequent immunological assays. Consistent with the DX enrichment, R1-EVs exhibited significantly higher antioxidant activity than ΔcrtI-EVs across multiple assays, including DPPH, ABTS, SOD, and CAT (Supplementary Figure S1).

### 3.2. R1-EVs Attenuate LPS-Induced DC Maturation Compared with ΔcrtI-EVs

#### 3.2.1. R1-EVs Suppress LPS-Induced Upregulation of Surface Costimulatory Molecules on the BMDCs More Effectively than ΔcrtI-EVs, Without Inducing Cytotoxicity

**Figure 2:**
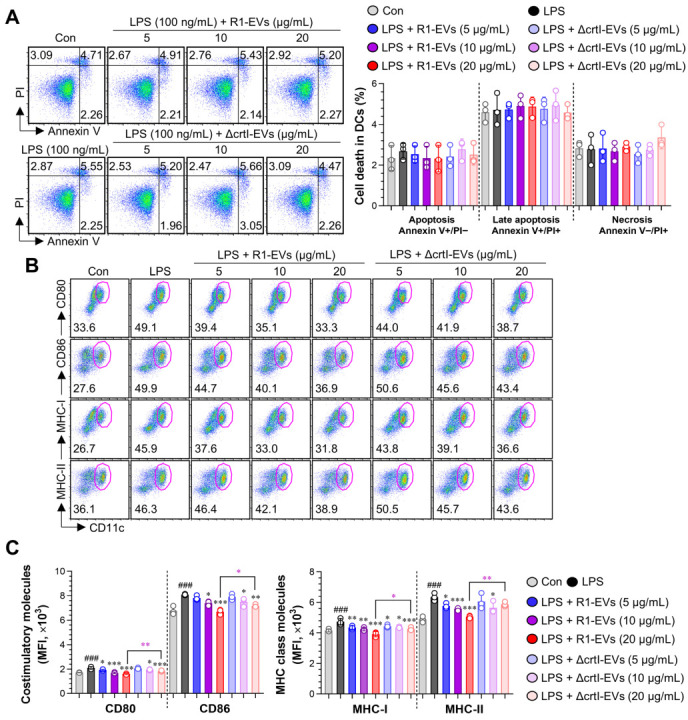
Cytotoxicity and surface maturation marker expression in the bone marrow-derived DC (BMDCs) treated with R1-EVs or ΔcrtI-EVs. (**A**) BMDCs were treated with R1-EVs or ΔcrtI-EVs at concentrations of 5, 10, and 20 μg/mL, LPS (100 ng/mL; positive control) or left untreated (control) for 18 h. The cell viability was assessed using Annexin V and propidium iodide (PI) staining to distinguish live, early apoptotic, and late apoptotic/necrotic cells. (**B**) For phenotypic maturation analysis, BMDCs were treated with LPS alone or in combination with R1-EVs or ΔcrtI-EVs (5, 10, and 20 μg/mL). After 18 h, CD11c^+^ BMDCs were analyzed by flow cytometry for surface expression of CD80, CD86, MHC-I, and MHC-II. (**C**) Expression levels of CD80, CD86, MHC-I, and MHC-II on CD11c^+^ BMDCs, presented as mean fluorescence intensity (MFI). The data represent three independent experiments (n = 3 per condition) and are expressed as mean ± SD. Statistical significance was determined using one-way ANOVA followed by Tukey’s post hoc test for multiple comparisons, and unpaired *t*-tests for selected pairwise comparisons (GraphPad Prism 10). ### *p* < 0.001 vs. control; * *p* < 0.05, ** *p* < 0.01, or *** p < 0.001 vs. LPS; * *p* < 0.05 or ** *p* < 0.01 between LPS + R1-EVs (20 μg/mL) and LPS + ΔcrtI-EVs (20 μg/mL).

The DC maturation is characterized by coordinated phenotypic changes, including upregulation of surface molecules (CD80, CD86, MHC-I, and MHC-II), increased secretion of pro-inflammatory cytokines, such as TNF-α and IL-12, and reduced endocytic capacity for antigen uptake [28,29]. These hallmarks serve as key indicators for assessing the immunological status of the DCs, and inhibition of these maturation-associated changes is often interpreted as a shift toward a more tolerogenic phenotype [30,31]. This shift is functionally associated with low expression of co-stimulatory molecules, impaired T cell activation, reduced pro-inflammatory cytokine production, and enhanced IL-10 secretion, which collectively contribute to the suppression of adaptive immune responses [32,33]. To investigate the immunomodulatory effects of R1-EVs and the potential contribution of the DX, we assessed the maturation status of the BMDCs under the LPS stimulation in the presence of either R1-EVs or ΔcrtI-EVs at concentrations of 5, 10, and 20 μg/mL. First, we evaluated cell viability using Annexin V and PI staining. The flow cytometry analysis revealed no evidence of apoptosis or necrosis in any of the treatment groups, including untreated cells, the LPS alone, or the LPS combined with either type of the EV (Figure 2A). In addition, no Annexin V^+^ or PI^+^ staining was detected in cells treated with R1-EVs or ΔcrtI-Evs alone, further supporting the absence of cytotoxicity). We next examined the expression of surface maturation markers on the BMDCs. The LPS stimulation markedly upregulated CD80, CD86, MHC-I, and MHC-II expression. Co-treatment with R1-EVs significantly suppressed this LPS-induced upregulation in a dose-dependent manner, while ΔcrtI-EVs exerted only a modest inhibitory effect (Figure 2B), suggesting that the presence of DX enhances the immunosuppressive activity of R1-EVs. Additionally, the EVs alone had no detectable effect on the expression of surface maturation markers (Supplementary Figure S2B). Collectively, these data show that R1-EVs attenuate the LPS-driven phenotypic maturation of the BMDCs more potently than ΔcrtI-EVs without inducing cytotoxicity, consistent with a DX-linked contribution.

#### 3.2.2. R1-EVs Suppress Pro-Inflammatory Cytokines, Induce IL-10 Secretion, and Preserve Antigen Uptake in LPS-Stimulated BMDCs More Effectively than ΔcrtI-EVs

**Figure 3:**
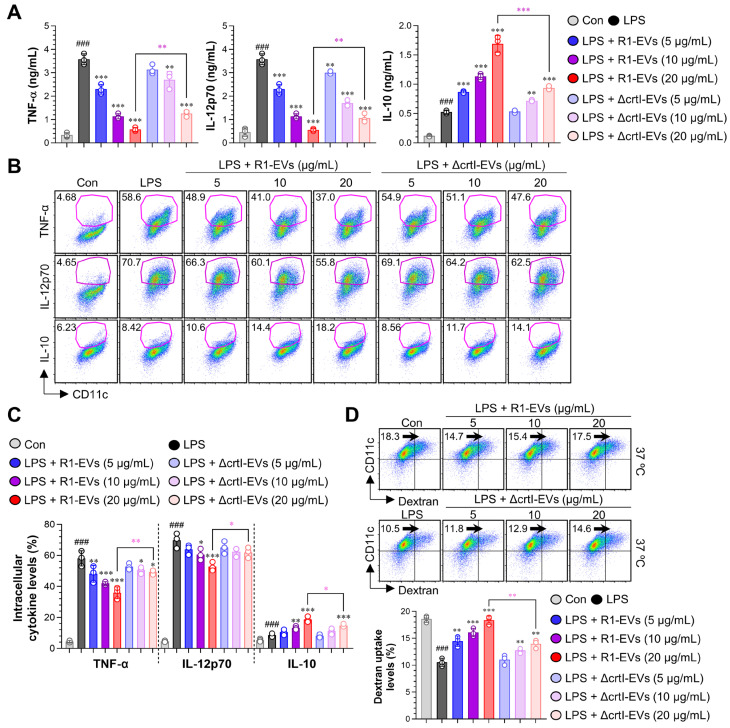
R1-EVs modulate cytokine secretion and preserve antigen uptake in the LPS-stimulated BMDCs. (**A**) The BMDCs were treated with the LPS (100 ng/mL) alone or co-treated with R1-EVs or ΔcrtI-EVs (5, 10, and 20 μg/mL) for 20 h. Supernatants were collected, and extracellular levels of TNF-α, IL-12p70, and IL-10 were measured by ELISA. (**B**) For intracellular cytokine analysis, BMDCs were treated under the same conditions in the presence of GolgiPlug for 8 h. Cells were stained for CD11c and intracellular TNF-α, IL-12p70, and IL-10, and analyzed by flow cytometry. (**C**) The intracellular cytokine-positive BMDCs expressed as the percentage of CD11c^+^ cells producing TNF-α, IL-12p70, or IL-10. (**D**) Antigen uptake was assessed by incubating treated BMDCs with FITC-dextran at 37 °C and 4 °C for 40 min, followed by staining with anti-CD11c and flow cytometric analysis. Data represent three independent experiments and are presented as mean ± SD (n = 3 per condition). Statistical significance was determined using one-way ANOVA followed by Tukey’s post hoc test for multiple comparisons, and unpaired *t*-tests for selected pairwise comparisons (GraphPad Prism 10). ### *p* < 0.001 vs. control; * *p* < 0.05, ** *p* < 0.01, or *** *p* < 0.001 vs. LPS; * *p* < 0.05, ** *p* < 0.01, or *** *p* < 0.001 between LPS + R1-EVs (20 μg/mL) and LPS + ΔcrtI-EVs (20 μg/mL), pink asterisks indicate significance for R1-EVs vs. ΔcrtI-EVs..

During the DC maturation, the secretion of pro-inflammatory cytokines such as TNF-α and IL-12 increases, promoting immune activation and T cell priming. In contrast, IL-10 plays a central role in establishing an anti-inflammatory and tolerogenic DC phenotype by limiting the expression of inflammatory mediators [33,34]. Therefore, a cytokine profile marked by reduced TNF-α/IL-12 and elevated IL-10 is widely regarded as an indicator of suppressed DC maturation and functional tolerance [35]. To assess how EVs affect cytokine secretion, the BMDCs were stimulated with the LPS (100 ng/mL) alone or co-treated with R1-EVs or ΔcrtI-EVs at 5, 10, and 20 μg/mL for 18 h. The ELISA analysis of culture supernatants showed that R1-EVs potently suppressed the production of pro-inflammatory cytokines TNF-α and IL-12p70, while significantly enhancing IL-10 levels compared to LPS alone. In contrast, ΔcrtI-Evs were less effective than R1-Evs in suppressing TNF-α and IL-12p70 secretion and exhibited a weaker enhancement of IL-10 production (Figure 3A). Consistently with previous findings from Supplementary Figure S2A,B, EV treatment alone did not alter cytokine production; however, it significantly increased extracellular IL-10, with R1-Evs inducing higher secretion than ΔcrtI-Evs (Supplementary Figure S2C). To validate these findings at the single-cell level, the intracellular cytokine staining was performed following 8 h of treatment in the presence of GolgiPlug. The flow cytometry analysis of CD11c^+^ BMDCs confirmed a decrease in intracellular TNF-α and IL-12p70 expression and a relative increase in IL-10 production upon R1-EV treatment, with weaker effects observed for ΔcrtI-EVs (Figure 3B). Because immature dendritic cells (DCs) exhibit high endocytic capacity, whereas maturation significantly reduces their ability to internalize exogenous antigens [36], we further assessed the functional status of the BMDCs by measuring FITC-dextran uptake capacity. The immature BMDCs were treated as above and then incubated with the FITC-dextran at 37 °C and 4 °C for 40 min. The LPS treatment significantly reduced FITC-dextran uptake, consistent with a mature DC phenotype. Co-treatment with R1-EVs increased dextran uptake compared to the LPS alone group, indicating retention of an immature, tolerogenic phenotype. In contrast, ΔcrtI-EVs–treated cells showed only partial increase in uptake, suggesting a diminished capacity to suppress the DC maturation relative to R1-EVs (Figure 3C). As a 4 °C control, dextran uptake was negligible and indistinguishable across all conditions, confirming the absence of active endocytosis and indicating that the differences observed at 37 °C reflect active uptake (Supplementary Figure S2D). These findings suggest that R1-EVs more effectively inhibit the LPS-induced DC maturation than the DX-deficient EVs, as reflected by reduced cytokine secretion and enhanced antigen uptake, thereby preserving the functional immaturity of the DCs.

### 3.3. R1-EVs Inhibit DC Maturation via Attenuation of MAPK and NF-κB Signaling Pathways

**Figure 4:**
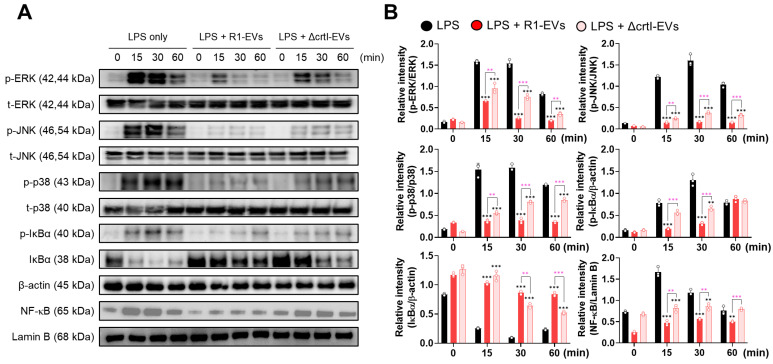
R1-EVs attenuate LPS-induced activation of MAPK and NF-κB signaling pathways and downstream DC maturation in a DX-dependent manner. (**A**) The BMDCs were stimulated with LPS (100 ng/mL) alone or co-treated with R1-EVs or ΔcrtI-EVs (20 μg/mL) for the indicated time points (0, 15, 30, and 60 min). Phosphorylation of ERK1/2, JNK, p38, and IκBα, as well as nuclear translocation of NF-κB p65, was assessed by immunoblotting. β-actin and lamin B were used as loading controls for cytosolic and nuclear fractions, respectively. (**B**) Densitometric analysis of immunoblots was performed, and signal intensities were normalized to β-actin (cytosolic proteins) or lamin B (nuclear proteins). All data are representative of three independent experiments and are presented as mean ± SD (n = 3 per group). Statistical significance was determined using one-way ANOVA followed by Tukey’s post hoc test for multiple comparisons, and unpaired *t*-tests for selected pairwise comparisons (GraphPad Prism 10). ** *p* < 0.01 or *** *p* < 0.001 vs. LPS; ** *p* < 0.01 or *** *p* < 0.001 between LPS + R1-EVs and LPS + ΔcrtI-EVs, pink asterisks indicate significance for R1-EVs vs. ΔcrtI-EVs.

The activation of MAPKs and NF-κB signaling cascades is known to play a pivotal role in the DC maturation in response to inflammatory stimuli such as LPS [37]. To investigate whether R1-EVs modulate these pathways, we performed time-course immunoblotting analyses for phosphorylated ERK1/2, JNK, p38, and IκBα, as well as nuclear translocation of NF-κB p65 in the BMDCs following the LPS stimulation. The LPS treatment rapidly induced phosphorylation of MAPKs and IκBα, along with increased nuclear accumulation of NF-κB p65. Co-treatment with R1-EVs markedly attenuated these LPS-induced signaling events in a time-dependent manner, indicating that R1-EVs suppress the activation of both MAPK and NF-κB pathways. In contrast, co-treatment with ΔcrtI-EVs, which lack the DX, resulted in markedly weaker attenuation of these pathways, indicating a DX-dependent contribution to the R1-EV effect (Figure 4A,B). These findings support the conclusion that R1-EVs attenuate the LPS-induced DC maturation by interfering with MAPK and NF-κB signaling pathways, and that DX is a key contributor to this immunomodulatory effect.

### 3.4. R1-EVs Attenuate Allogeneic T Cell Responses by Modulating DC–T Cell Interactions

**Figure 5:**
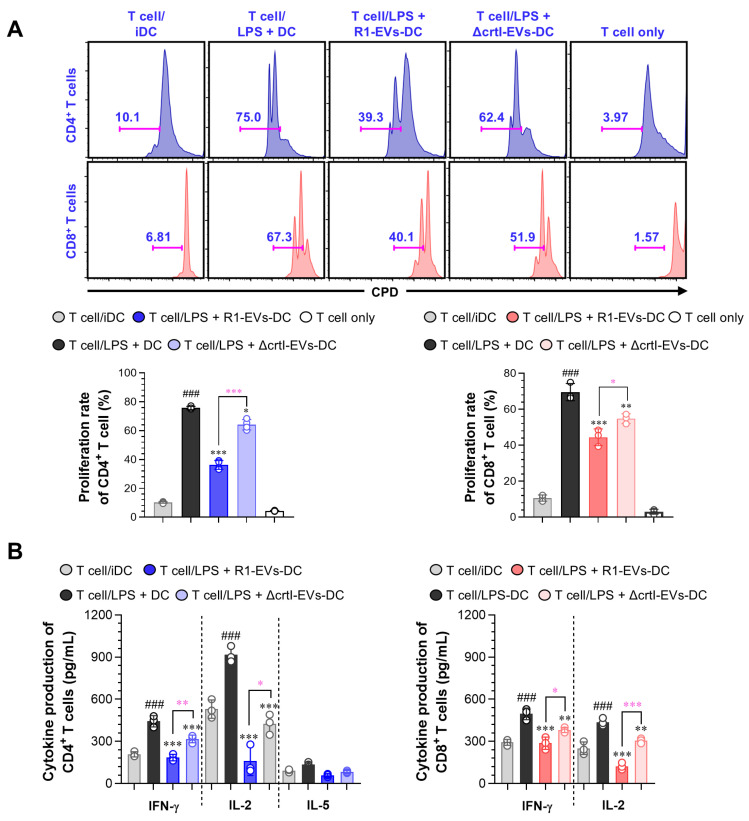
R1-EV–treated DCs suppress allogeneic T cell proliferation and cytokine production more effectively than ΔcrtI-EVs-EV–treated cells. (**A**) The BMDCs were pretreated with LPS (100 ng/mL), R1-EVs (20 μg/mL), or ΔcrtI-EVs (20 μg/mL), alone or in combination, for 18 h, and then co-cultured with Cell Proliferation Dye (CPD)–labeled allogeneic CD4^+^ and CD8^+^ T cells for 3 days in a mixed lymphocyte reaction (MLR) assay. The T cell proliferation was assessed by flow cytometry based on CPD dilution. (**B**) The culture supernatants from MLR assays were collected, and cytokine levels were quantified by ELISA to assess T cell polarization: Th1-type cytokines (IFN-γ, IL-2) and Th2-type cytokine (IL-5). The data are representative of three independent experiments and are presented as mean ± SD (n = 3 per condition). Statistical significance was determined using one-way ANOVA followed by Tukey’s post hoc test for multiple comparisons, and unpaired *t*-tests for selected pairwise comparisons (GraphPad Prism 10). ### *p* < 0.001 vs. T cell/iDC; * *p* < 0.05, ** *p* < 0.01, or *** *p* < 0.001 vs. T cell/LPS + DC; * *p* < 0.05, ** *p* < 0.01, or *** *p* < 0.001 between T cell/LPS + R1-EVs-DC and T cell/LPS + ΔcrtI-EVs-DC, pink asterisks indicate significance for R1-EVs vs. ΔcrtI-EVs.

The R1-EVs-treated BMDCs displayed anti-inflammatory activity by changing to a tolerogenic phenotype in response to LPS stimulation. The DCs bridge innate and adaptive immunity by presenting antigens, co-stimulatory molecule interaction, and secreting cytokines that guide T cell activation and differentiation [38]. IL-10–producing tolerogenic DCs are known to suppress the T cell proliferation by limiting co-stimulatory and inflammatory signals [39,40]. We therefore evaluated whether R1-EV–treated BMDCs could regulate allogeneic T cell responses in a mixed lymphocyte reaction assay. The BMDCs were pretreated with LPS (100 ng/mL) alone or in combination, and subsequently co-cultured with allogeneic CD4^+^ and CD8^+^ T cells labeled with a cell proliferation dye (CPD) eFluor 450. T cells co-cultured with BMDCs treated with LPS alone exhibited robust proliferation, while those co-cultured with R1-EV/LPS–treated BMDCs showed markedly reduced proliferation of both CD4^+^ and CD8^+^ T cells. Notably, this effect was diminished when BMDCs were treated with ΔcrtI-EVs and LPS, indicating that the presence of DX in R1-EVs contributes to their immunomodulatory function (Figure 5A). To further evaluate the functional consequence of suppressed the T cell proliferation, we measured cytokine production in the culture supernatants. Levels of Th1 (IFN-γ, IL-2) and Th2 (IL-5) cytokines were elevated in T cells co-cultured with LPS-stimulated BMDCs, while the secretion of these cytokines was significantly attenuated in the R1-EV/LPS group (Figure 5. Consistent with these findings, the ΔcrtI-EVs-EV/LPS group exhibited reduced suppressive capacity compared to the R1-EV/LPS group. Together, these data suggest that R1-EVs attenuate the capacity of the LPS-stimulated BMDCs to activate allogeneic T cells, highlighting the pivotal role of DX in modulating the DC–T cell interactions under inflammatory conditions.

### 3.5. IL-10 Neutralization Reverses R1-EV–Mediated Tolerogenic Phenotypes in Dendritic Cells and T Cells

**Figure 6:**
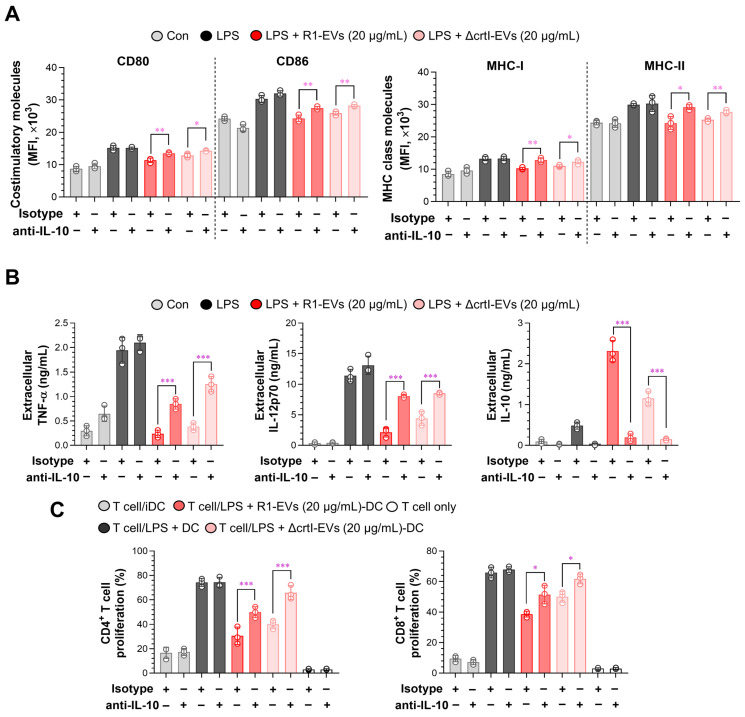
IL-10 neutralization reverses the tolerogenic effects of LPS/R1-EV–treated BMDCs. BMDCs were pretreated with anti-IL-10 monoclonal antibody (5 ng/mL) or rat IgG isotype control for 2 h prior to stimulation with the LPS in combination with R1-EVs or ΔcrtI-EVs. (**A**) Surface expression of CD80, CD86, MHC-I, and MHC-II on CD11c⁺ cells was analyzed by flow cytometry. Data represent mean fluorescence intensity (MFI). (**B**) Levels of TNF-α, IL-12p70, and IL-10 in culture supernatants were quantified by ELISA. (**C**) CD4^+^ and CD8^+^ T cells isolated from BALB/c splenocytes were labeled with a cell proliferation dye and co-cultured with pretreated BMDCs under the indicated conditions. T cell proliferation was analyzed by flow cytometry. The data are representative of three independent experiments and are presented as mean ± SD (n = 3 per condition). Statistical significance was determined using unpaired *t*-tests for pairwise comparisons (GraphPad Prism 10). * *p* < 0.05, ** *p* < 0.01, or *** *p* < 0.001 between T cell/LPS + R1-EVs-DC and T cell/LPS + ΔcrtI-EVs-DC.

Given that IL-10 is a central negative regulator of DC activation and tissue inflammation [41], we tested its requirement for the tolerogenic activity of R1-EVs by performing neutralization experiments with an anti-IL-10 monoclonal antibody. The BMDCs were pretreated with anti-IL-10 or rat IgG1 isotype control for 2 h prior to stimulation with LPS combined with either R1-EVs or ΔcrtI-EVs. Neutralization of IL-10 significantly restored the surface expression of CD80, CD86, MHC-I and MHC-II in BMDCs compared to isotype-treated controls (Figure 6A), indicating reversal of the maturation-suppressive effect by R1-EVs. Consistently, IL-10 neutralization abolished the R1-EV–mediated suppression of TNF-α and IL-12p70 and reduced extracellular IL-10 to near-baseline levels, confirming effective blockade (Figure 6B). We next assessed whether IL-10 is also required for the suppression of T cell activation by R1-EV–treated BMDCs. In allogeneic mixed lymphocyte reactions, the proliferation of both CD4^+^ and CD8^+^ T cells was significantly restored when co-cultured with IL-10–neutralized BMDCs treated with LPS + R1-EVs or LPS + ΔcrtI-EVs (Figure 6C). These results collectively demonstrate that IL-10 is essential for the induction of the tolerogenic phenotype by R1-EVs, affecting DC maturation, pro-inflammatory cytokine production, and downstream T cell responses.

## 4. Discussion

Here we show that the DX-enriched EVs from *D. radiodurans* (R1-EVs) re-program the DCs toward a tolerogenic state under inflammatory challenge. R1-EV–exposed DCs down-regulate co-stimulatory and the MHC molecules, reduced production of pro-inflammatory cytokines, sustain antigen uptake, and elicit reduced allogeneic T-cell activation under LPS challenge. Mechanistically, R1-EVs attenuate the LPS-triggered MAPK and NF-κB signaling, and IL-10 neutralization reverses these effects, indicating a primarily IL-10–dependent pathway. EVs from a DX-deficient mutant (ΔcrtI-EVs) are less active, implicating the carotenoid DX as a contributing determinant. Together, these findings suggest that bacteria-derived nanovesicles can instruct DC tolerance and broaden the functional scope of carotenoids beyond antioxidant protection to immune modulation.

The DCs orchestrate the initiation and quality of adaptive responses through three coordinated “signals”: peptide–MHC presentation to the TCR (signal 1), costimulation (CD80 and CD86; signal 2), and polarizing cytokines (signal 3) that imprint T-cell fate [38,42]. Tolerogenic DCs represent a counter-state characterized by dampened costimulation and pro-inflammatory cytokines, preserved endocytosis, and production of immunoregulatory mediators such as IL-10, thereby limiting effector priming and fostering peripheral tolerance [40,43,44]. In our study, R1-EV exposure under the LPS challenge reduced surface maturation markers (Figure 2B,C) and pro-inflammatory cytokines (Figure 3A–C) while preserving antigen uptake (Figure 3D), in concert with attenuation of MAPK and NF-κB activation (Figure 4). These findings situate R1-EVs as active modulators of the DC activation set-point rather than passive antioxidants. Notably, the observation that R1-EVs increased IL-10 production provides a mechanistic bridge to an IL-10–dependent tolerogenic axis considered below (Figure 3A–C; Supplementary Figure S2C).

IL-10 is a central negative regulator of dendritic-cell activation, suppressing NF-κB/MAPK–dependent transcriptional programs, reducing IL-12p70, and stabilizing a low-costimulation phenotype that limits Th1/Th17 priming while promoting regulatory networks [45,46]. Consistent with this central role, IL-10–centered tolerance reliably dampens tissue inflammation across diverse settings [47,48]: *Pediococcus pentosaceus* KF159 mitigated house-dust-mite–induced atopic dermatitis by boosting IL-10 and regulatory T cells (Tregs) induction [49], and helminth-derived metabolites imprinted DCs with tolerogenic functional, metabolic, and transcriptional signatures that attenuated experimental colitis [50]. In keeping with this tolerogenic shift, R1-EV–conditioned DCs exhibited a diminished capacity to prime allogeneic T cells, as evidenced by reduced proliferation of CD4^+^ and CD8^+^ subsets and decreased IFN-γ/IL-2 production in MLR (Figure 5). Importantly, neutralization of IL-10 significantly abrogated the R1-EV–mediated suppression of DC maturation and T-cell responses (Figure 6), highlighting IL-10 as a key effector of R1-EV tolerogenicity. Together, these lines of evidence support the view that R1-EVs exert tolerogenic activity via an IL-10–dependent axis.

Carotenoids are renowned ROS-scavengers with membrane-stabilizing, lipid-peroxidation–limiting, and anti-inflammatory properties that secondarily modulate innate signaling thresholds [51,52]. In line with this paradigm, astaxanthin and fucoxanthin attenuate LPS-driven macrophage inflammation by suppressing NF-κB and MAPK signaling, thereby lowering iNOS/COX-2 and inflammatory mediators (NO, PGE_2_, TNF-α, and IL-1β), and in some models increasing antioxidant enzymes (SOD and CAT) [53,54]. Although the superior antioxidant capacity of R1-EVs compared with ΔcrtI-EVs (Supplementary Figure S1) likely contributes to suppression of oxidative stress–sensitive signaling such as MAPK and NF-κB, this notion is supported by precedent: β-carotene has been reported to attenuate LPS-induced activation of NF-κB, JNK, and p38 MAPK in macrophages, while simultaneously inhibiting ROS accumulation and preventing NF-κB p65 nuclear translocation through stabilization of IκBα [55,56]. These findings consolidate the perspective that the antioxidant function of R1-EVs may likewise impair LPS-driven proinflammatory signaling cascades. *D. radiodurans* forms pink-to-red colonies owing to oxygenated carotenoids (xanthophylls); its major pigment, deinoxanthin—a hydroxylated ketocarotenoid—contributes to oxidative and radiation-stress resistance, alongside other cellular defenses [12]. Consistent with carotenoid-dependent pigmentation, the R1-EV fraction exhibited a pink hue whereas ΔcrtI-EVs lacked visible coloration (Figure 1A), supporting DX enrichment in R1-EVs. In this work, we directly tested DX’s contribution by comparing wild-type R1-EVs with EVs from a DX-deficient mutant. ΔcrtI-EVs were consistently less effective than R1-EVs at enforcing DC tolerance, reflected by weaker attenuation of maturation, the MAPK and NF-κB signaling, and downstream T-cell activation (Figure 2, Figure 3, Figure 4 and Figure 5). Notably, IL-10 induction was also attenuated with ΔcrtI-EVs, further implicating DX in the tolerogenic program.

While these results define an IL-10– and DX-dependent framework for R1-EV–mediated tolerance, several limitations temper our interpretation and outline priorities for future studies. First, ΔcrtI-EVs retained partial tolerogenic activity in our main readouts—maturation markers, cytokines (including weaker but present IL-10), antigen uptake, signaling, and MLR—indicating that factors are beyond the DX contribution. Moreover, ΔcrtI-EVs also exhibited partial antioxidant activity ), suggesting that other vesicular components, such as enzymes, lipoproteins, or small RNAs, may participate in both immunomodulatory and redox-protective effects. To systematically uncover these contributors, activity-guided fractionation integrated with lipidomics, proteomics, metabolomics, and transcriptomic/small RNA profiling will be applied in future studies. Second, our analyses were conducted in vitro, which limits direct inference to disease contexts. Although prior work showed increased Treg frequencies after R1-EV administration in a total-body irradiation model [21], disease-relevant in vivo validation is needed in inflammatory and autoimmune settings such as DSS colitis, experimental autoimmune encephalomyelitis (EAE), and allergic airway inflammation, as well as in antigen-specific systems, such as OVA/OT-II, to define efficacy, dose–response, durability, and specificity. Building on our previous in vivo findings, we plan to extend these investigations into such disease-relevant models to strengthen the translational relevance of R1-EVs. Third, the upstream determinants of IL-10 induction remain unresolved. Receptor and pathway mapping—using antagonists of the TLRs and other pattern-recognition receptors, DC-intrinsic genetics, redox-pathway perturbation, and inhibitors of clathrin-mediated endocytosis, dynamin-dependent uptake, and macropinocytosis—should localize the initiating nodes; in parallel, phenocopy with recombinant IL-10 and in vivo IL-10R blockade will refine necessity and sufficiency. Fourth, causal attribution to the DX and experimental standardization require further rigor, such as genetic complementation or DX reconstitution, per-particle DX quantification by LC–MS, particle- and protein-normalized dosing, quantification of residual pathogen-associated molecular patterns and lipoproteins, cargo-depletion controls, and batch-to-batch stability assessments to strengthen the DX-dependent interpretation.

Beyond these mechanistic considerations, several practical aspects merit attention for translational application. The EV doses used in this study (up to 20 μg/mL protein, ~6 × 10^9^ particles/mL) are consistent with concentrations commonly applied in bacterial and plant EV studies [57,58]. Cytokine suppression plateaued beyond this level in our screening, supporting its use as the maximal dose. Furthermore, in vivo administration at 10 mg/kg (~6 × 10^10^ particles per 20 g mouse) was previously shown to be safe without organ toxicity [21]. Future pharmacokinetic and dose-response analyses will be essential to determine relevance in vivo. Furthermore, we did not directly test the DX alone; while our focus was to examine DX within its native vesicular context, purified DX experiments will be informative to distinguish a vesicle-dependent activity from a free-carotenoid, and future studies will also explore strategies to reconstitute or load the DX into EVs to optimize delivery and rigorously validate the DX-dependent contributions. In addition, direct quantification of the DX within R1-EVs by LC–MS or the HPLC was not performed in this study. Instead, we inferred the DX enrichment from both the visible pigmentation of R1-EVs and the consistently weaker activity of ΔcrtI-EVs across functional assays. Supporting proteomic data from our group’s previous work, the authors of [21] further revealed high abundance of S-layer proteins, including Hpi, which are known in *D. radiodurans* to form the S-layer deinoxanthin-binding complex (SDBC) that stabilizes carotenoids [59,60]. These indirect lines of evidence strengthen the interpretation that R1-EVs are enriched in the DX, although future studies will incorporate an LC–MS/HPLC-based quantification to rigorously validate this. Finally, biosafety concerns must be addressed, as bacterial EVs may contain immunogenic or endotoxin-like molecules. Although our preparations were carefully purified, additional methods, such as size-exclusion chromatography, density-gradient ultracentrifugation, and enzymatic depletion of bacterial lipoproteins, may further improve safety. Preclinical studies will be needed to evaluate potential off-target immune activation.

## 5. Conclusions

R1-EVs re-program the DCs toward a tolerogenic state under inflammatory challenge, attenuating the MAPK/NF-κB activation and limiting downstream T-cell priming. The tolerogenic activity proceeds through an IL-10–dependent axis, as IL-10 neutralization reverses the R1-EV effect, and involves the DX as a major contributor, given the consistently weaker activity of ΔcrtI-EVs. In addition, R1-EVs displayed significantly stronger antioxidant capacity than ΔcrtI-EVs, consistent with the DX enrichment, indicating that their redox-protective activity may further contribute to the suppression of inflammatory signaling. To our knowledge, this is the first evidence that a bacterial carotenoid contributes to tolerogenic programming of the DCs, extending carotenoid biology beyond antioxidant protection to active immune modulation. These findings position bacteria-derived EVs as immunomodulatory platforms, with IL-10 and DX serving as tunable levers to control the DC activation. Priority next steps include in vivo validation in disease-relevant models and identification of additional bioactive cargo that collaborates with the DX to enforce tolerance.

## Data Availability

The datasets generated and analyzed during the current study are included in this published article and its Supplementary Materials. Further inquiries can be directed to the corresponding author.

## Supplementary Materials

The following supporting information can be downloaded at: https://www.mdpi.com/article/10.3390/antiox14091108/s1, Figure S1: Antioxidant activities of R1-EVs and ΔcrtI-EVs;  Figure S2: Effects of R1-EVs and ΔcrtI-EVs on BMDC viability, surface maturation marker expression, cytokine secretion, and antigen uptake; Table S1: Primers used in this study; Supplementary Methods: Antioxidant activity assays. 

## Abbreviations

The following abbreviations are used in this manuscript:

ABTS	2,2′-Azino-bis (3-ethylbenzothiazoline-6-sulfonic acid)
BEVs	bacterial EVs
BMDCs	bone marrow-derived DCs
CAT	catalase
DC	dendritic cell
DLS	dynamic light scattering
DPPH	2,2-Diphenyl-1-picrylhydrazyl
DX	deinoxanthin
EVs	Extracellular vesicles
FBS	fetal bovine serum
IL	interleukin
LPS	lipopolysaccharide
MAPK	mitogen-activated protein kinase
MFI	mean fluorescence intensity
MHC	major histocompatibility complex
MLR	mixed lymphocyte reaction
NF-κB	Nuclear Factor kappa-light-chain-enhancer of activated B cells
NTA	nanoparticle tracking analysis
OMVs	outer membrane vesicles
PDI	polydispersity index
PI	propidium iodide
R1-EVs	D. radiodurans–derived EVs
rmGM	recombinant mouse granulocyte-macrophage
CSF	colony-stimulating factor
ROS	reactive oxygen species
RT	room temperature
SD	standard deviation
SDBC	S-layer deinoxanthin-binding complex
SOD	superoxide dismutase
TEM	transmission electron microscopy
TFF	tangential flow filtration
TGY	tryptone glucose yeast extract
