# Supplementary material for: Deinoxanthin-Enriched Extracellular Vesicles from Deinococcus radiodurans Drive IL-10–Dependent Tolerogenic Programming of Dendritic Cells
Source: Antioxidants (Basel). 2025 Sep 12;14(9):1108. doi: 10.3390/antiox14091108 (PMC12466609; doi:10.3390/antiox14091108)
Supplement: Supplementary file 1 [file antioxidants-14-01108-s001.zip › antioxidants-3843191-supplementary.pdf]

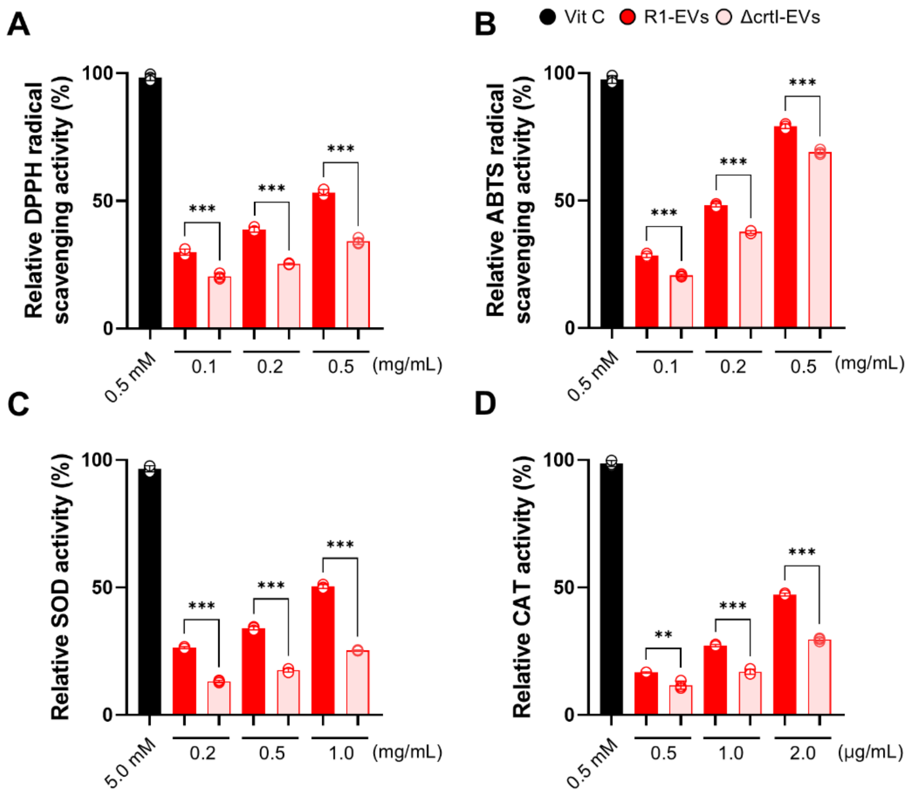

Supplementary Figure S1. Antioxidant activities of R1-EVs and ΔcrtI-EVs**.** The antioxidant potential of extracellular vesicles (EVs) derived from *Deinococcus radiodurans* wild-type (R1-EVs) and carotenoid-deficient mutant (ΔcrtI-EVs) was evaluated using radical scavenging and antioxidant enzyme activity assays. Vitamin C (Vit C) was used as a positive control in all assays (0.5 mM for DPPH, ABTS, and CAT; 5.0 mM for SOD). (A) 2,2-Diphenyl-1-picrylhydrazyl (DPPH) radical scavenging activity of R1-EVs and ΔcrtI-EVs at 0.1, 0.2, and 0.5 mg/mL. (B) 2,2′-Azino-bis(3-ethylbenzothiazoline-6-sulfonic acid) (ABTS) radical scavenging activity under the same conditions. (C) Superoxide dismutase (SOD) activity of EVs at 0.2, 0.5, and 1 mg/mL. (D) Catalase (CAT) activity of EVs at 0.5, 1, and 2 μg/mL. All activities are presented as relative activity (%) normalized to positive control (Vit C). All data are representative of three independent experiments and are presented as mean ± SD (n = 3 per group). ***p < 0.01 or ***p < 0.001* between R1-EVs and ΔcrtI-EVs.

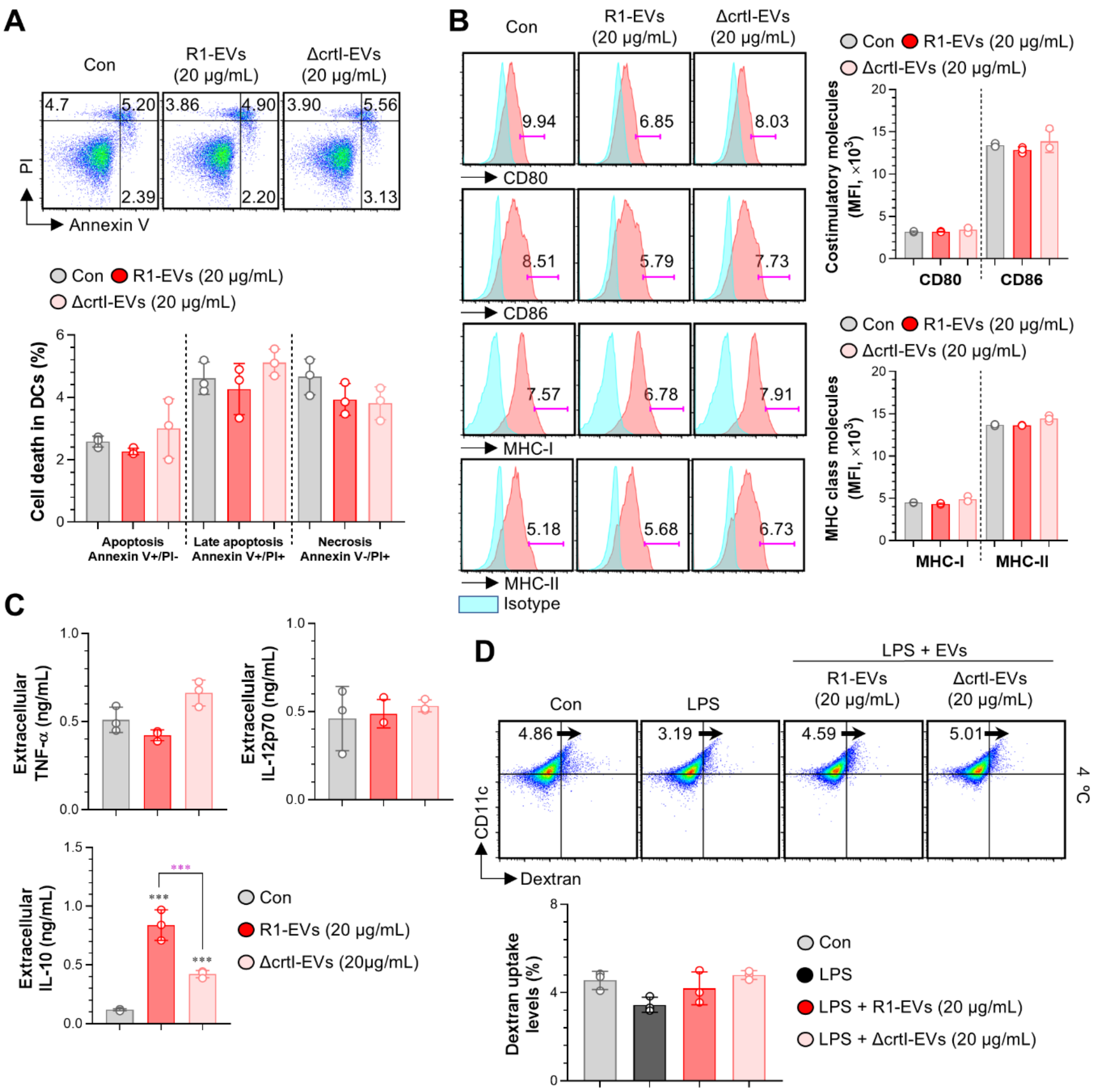

Supplementary Figure S2. Effects of R1-EVs and ΔcrtI-EVs on BMDC viability, surface maturation marker expression, cytokine secretion, and antigen uptake. (A) Annexin V/PI staining of BMDCs treated with R1-EVs or ΔcrtI-EVs (20 μg/mL) for 18 h, showing no detectable apoptosis or necrosis compared with untreated controls. (B) Flow cytometry histograms of CD80, CD86, MHC-I, and MHC-II expression in BMDCs treated with EVs alone for 18 h, indicating no upregulation of maturation markers. (C) ELISA quantification of TNF-α, IL-12p70, and IL-10 in culture supernatants from BMDCs treated with EVs alone for 18 h, showing no significant changes in TNF-α and IL-12p70 production, but a significant increase in IL-10 secretion compared with untreated controls. (D) FITC–dextran uptake assay in BMDCs stimulated with LPS (100 ng/mL) alone or co-treated with R1-EVs or ΔcrtI-EVs (20 μg/mL) for 18 h, followed by incubation with FITC–dextran at 4 °C for 40 min. Uptake was negligible across all conditions, confirming the absence of active endocytosis at low temperature.

Table S1. Primers used in this study

Name	Sequence (5′ to 3′)[1]	Description
crtI-DD-UF	CAAACTCGAGGAAGGCGAG	*crtI deletion*
crtI-DD-UR	GACTTCCGAGGTTTACTGTC**<u>CCCGGG</u>**GT CATACGGATTCCGCTTAA	*crtI deletion*
crtI-DD-DF	TTAAGCGGAATCCGTATGAC**<u>CCCGGG</u>**G ACAGTAAACCTCGGAAGTC	*crtI deletion*
crtI-DD-DR	ATGTCGGTGTCGACTTCGG	*crtI deletion*

[1]Tags with restriction sites are in boldface characters and underlined

Supplementary Methods

1. Antioxidant Activity Assays

The antioxidant activities of extracellular vesicles (EVs) derived from *Deinococcus radiodurans* wild-type (R1-EVs) and carotenoid-deficient mutant (ΔcrtI-EVs) were evaluated using 2,2-diphenyl-1-picrylhydrazyl (DPPH) radical scavenging, 2,2′-azino-bis(3-ethylbenzothiazoline-6-sulfonic acid) (ABTS) radical scavenging, catalase (CAT) activity, and superoxide dismutase (SOD) activity assays. Vitamin C (Vit C) was used as the positive control in all assays (0.5 mM for DPPH, ABTS, and CAT; 5.0 mM for SOD).

1.1. DPPH Radical Scavenging Assay

DPPH radical scavenging activity was assessed using the 0.2 mM DPPH dissolved in MeOH. R1-EVs and ΔcrtI-EVs were prepared at concentrations of 0.1, 0.2, and 0.5 mg/mL. Briefly, 100 μL of EV solution was mixed with 100 μL of DPPH working solution in a 96-well plate and incubated at room temperature for 30 min in the dark. The absorbance was measured at 517 nm using a microplate reader (Infinite 200Pro, Tecan, Männedorf, Switzerland).

1.2. ABTS Radical Scavenging Assay

ABTS radical scavenging activity was assessed using the $ABTS^+$ solution prepared by mixing 7 mM ABTS with 2.45 mM potassium persulfate ($K_2S_2O_8$) and incubating the mixture in the dark at room temperature for 12–16 h. R1-EVs and ΔcrtI-EVs were prepared at concentrations of 0.1, 0.2, and 0.5 mg/mL. For the assay, 20 μL of EV solution was added to 180 μL of $ABTS^+$ working solution in a 96-well plate and incubated for 10 min at room temperature. The absorbance was measured at 734 nm using a microplate reader (Infinite 200Pro, Tecan)..

1.3. Superoxide Dismutase (SOD) Activity Assay

SOD activity was assessed using the EZ-SOD assay kit (DG-SOD400; DoGenBio). EVs were prepared at 0.2, 0.5, and 1 mg/mL. 20 μL of EV solution was mixed with 200 μL of WST-1 working solution and 20 μL of enzyme working solution. The reaction mixture was incubated at 37 °C for 20 min, and the absorbance was measured at 450 nm using a microplate reader (Infinite 200Pro, Tecan).

1.4. Catalase (CAT) Activity Assay

CAT activity was assessed using the EZ-Catalase assay kit (DG-CAT400; DoGenBio). EVs were prepared at 0.5, 1, and 2 μg/mL. In each well, 25 μL of EV solution was mixed with 25 μL of hydrogen peroxide (H_2O_2) substrate solution and incubated at room temperature for 30 min. Then, 50 μL of Oxi-Probe/HRP working solution was added and incubated at 37 °C for 30 min. The absorbance was measured at 560 nm using a microplate reader (Infinite 200Pro, Tecan).
